# Learning what to approach

**DOI:** 10.1371/journal.pbio.3000043

**Published:** 2018-10-11

**Authors:** Neir Eshel, Elizabeth E. Steinberg

**Affiliations:** Department of Psychiatry and Behavioral Sciences, Stanford University, Stanford, California, United States of America

## Abstract

Most decisions share a common goal: maximize reward and minimize punishment. Achieving this goal requires learning which choices are likely to lead to favorable outcomes. Dopamine is essential for this process, enabling learning by signaling the difference between what we expect to get and what we actually get. Although all animals appear to use this dopamine prediction error circuit, some do so more than others, and this neural heterogeneity correlates with individual variability in behavior. In this issue of *PLOS Biology*, Lee and colleagues show that manipulating a simple task parameter can bias the animals’ behavioral strategy and modulate dopamine release, implying that how we learn is just as flexible as what we learn.

Learning the value of objects in the environment is critical for survival. Some mushrooms are deadly; others provide sustenance. Rain in the dry season can be a harbinger of life, while in a monsoon, it means heading for cover. How do animals learn these values and adapt them over time? One of the most common ways is through trial and error. As we explore our environment, we make predictions about the value of stimuli around us. If outcomes match our predictions, there is no need to adapt. If outcomes are different, we use that discrepancy to improve our predictions for the future. This difference between actual and expected outcome is known as prediction error, and it turns out to be crucial for learning in animals [1–3] as well as machines [4,5]. As befitting such a conserved learning mechanism, the brain has developed a fine-tuned system to encode it.

In the 1990s, Schultz and colleagues [6,7] recorded monkey dopamine (DA) neurons and discovered a curious response. When monkeys received an unexpected reward, such as a drop of juice, DA neurons became excited. If animals received the same reward but fully expected it, DA neurons showed no response. Instead, DA neurons fired to the earliest reliable predictor of the reward, typically a sound or picture that indicated juice was coming soon. Finally, if the reward was expected but never materialized, DA neurons dipped below their baseline firing rate at precisely the moment when the reward was anticipated. Together, these results imply that DA neurons encode the difference between actual and expected reward—in other words, reward prediction error (RPE), the precise signal already known to facilitate learning.

The link between DA and RPE has been replicated and extended numerous times in a host of species and tasks (for review see [8]). Notably, modern neuroscience tools have confirmed that prediction error neurons are indeed dopaminergic [9] and have revealed a local circuit in the ventral tegmental area that is partially responsible for calculating the RPE signal [10]. Furthermore, causal manipulations have demonstrated that both positive [11] and negative [12] DA RPEs are sufficient to cause associative learning.

While RPE signaling may represent the dominant population response [13], as more investigators began to study DA and reinforcement learning, it became apparent that DA neurons also encoded other signals. In rats trained to associate an audio–visual stimulus (insertion of a metal lever) with the delivery of a food pellet into a nearby receptacle, an intriguing relationship was found between heterogeneity in DA signaling and individual variability in behavior (Fig 1) [14]. Some rats, termed “sign-trackers,” readily approached and engaged with the reward-predictive lever cue, even though this behavior had no effect on subsequent reward delivery. Other rats, termed “goal-trackers,” used the lever only to time entry into the receptacle in anticipation of food; otherwise, they ignored the lever. Strikingly, these behavioral strategies were characterized by distinct DA responses: DA RPE signals were observed only in sign-trackers. For goal-trackers, cue-evoked DA release was weak, and the DA response to expected reward failed to decline, even when the rats had clearly learned the cue–reward association. Furthermore, pharmacological blockade of DA signaling disrupted the sign-tracking response but had no effect on goal tracking. These data suggest that individual differences in allocating attention and attributing value to meaningful stimuli may be driven, at least in part, by endogenous variation in the function of midbrain DA neurons.

**Fig 1:**
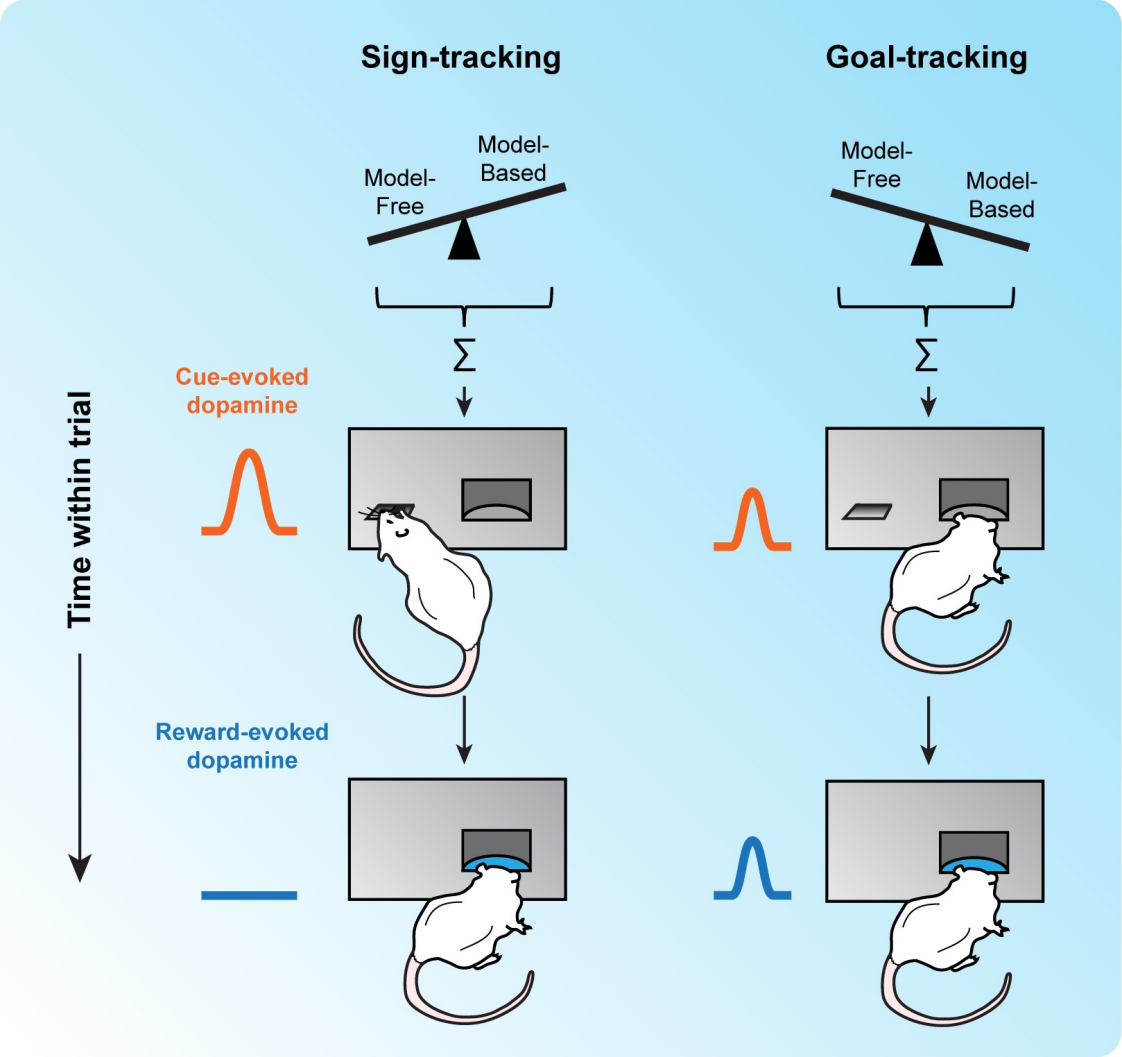
Behavioral variability and DA response. Hungry rats perform a task in which the appearance of a lever predicts food delivery several seconds later. When the lever is presented, some rats, termed sign-trackers (left), immediately approach the lever, while other rats, termed goal-trackers (right), approach the food cup instead. When reward is delivered, all rats approach the food cup. Previous work has demonstrated different patterns of DA release for these two groups: sign-trackers exhibit large DA release to the lever but not the reward, while goal-trackers show small but persistent DA responses to the lever and the reward. Both the behavioral and the neural differences between these groups have been interpreted to reflect the relative weights of two learning systems: model free and model based (see text). When model-free learning predominates, rats sign track; when model-based learning predominates, they goal track. DA, dopamine.

The empirical finding that stimuli can acquire different motivational properties in individuals trained on the same task is difficult to explain computationally with DA RPEs. To address this problem, Lesaint and colleagues [15] recently proposed a model that builds on previous efforts in two important ways. Typically, reinforcement learning algorithms use RPEs to drive learning about the value of states [16], not discrete stimuli. The Sign-Tracking and Goal-Tracking (STGT) model instead uses RPEs to create factored representations in which the value of stimuli can be adjusted independently, enabling the lever and food cup to acquire distinct motivational properties.

The second key advance is that values calculated by multiple learning systems are integrated as a weighted sum instead of relying exclusively on a value derived from RPEs. Given the wide range of situations that humans and other animals encounter in complex, unpredictable environments, it is unlikely that a single learning system can optimally address all potential problems. The STGT model leverages two previously described systems, termed model free and model based [17,18]. Model-free systems learn the value of actions and events through trial and error and store this information for later use. When the same stimuli are encountered again, past experience serves as a guide to estimate future outcomes. In contrast, model-based systems draw on an internal model of the world to make cognitive predictions of future events by forward inference. Model-based systems are able to flexibly generate goal-directed choices based on prospective assessment of the consequences of events or actions, without those consequences actually having to be experienced. Model-free and model-based learning systems offer complementary advantages and are thought to be implemented by distinct neural circuits [19]. Notably, DA RPEs are ideally suited to drive model-free learning.

The STGT model integrates stimulus values computed by model-free and model-based systems, with individual variation determining the weight assigned to each component. For sign-trackers, the DA-dependent model-free system dominates, assigning greater value to the lever that perfectly predicts reward. Goal-trackers preferentially rely on the DA-independent model-based system, which infers the optimal behavior to maximize reward and favors approach to the location where it will appear. Intermediate behavioral phenotypes are easily explained as a balanced weighting of values computed by the two systems.

In this issue of *PLOS Biology*, Lee and colleagues [20] test a specific hypothesis from the STGT model: changing the intertrial interval (ITI) will modulate DA signaling and bias animals between sign tracking and goal tracking (Fig 2). The logic goes like this: the longer the ITI, the more times an animal might visit the food cup between trials, when there is no food available. Each time this happens, DA neurons encode a negative RPE, and the value of the food cup goes down. Eventually, the food cup loses its salience to the animal, and the animal becomes more likely to sign-track, approaching the lever rather than the food cup. After all, the lever is more reliable at signaling reward: it always predicts food delivery, while the food cup often does not. Mirroring the behavior, DA neurons might show large bursts of activity both when the lever is presented—denoting the lever’s increased value to the animal—and when the food cup actually contains food, since this has become surprising. All of these predictions are reversed if the ITI is short and the animal has limited opportunity to visit an empty food cup.

**Fig 2:**
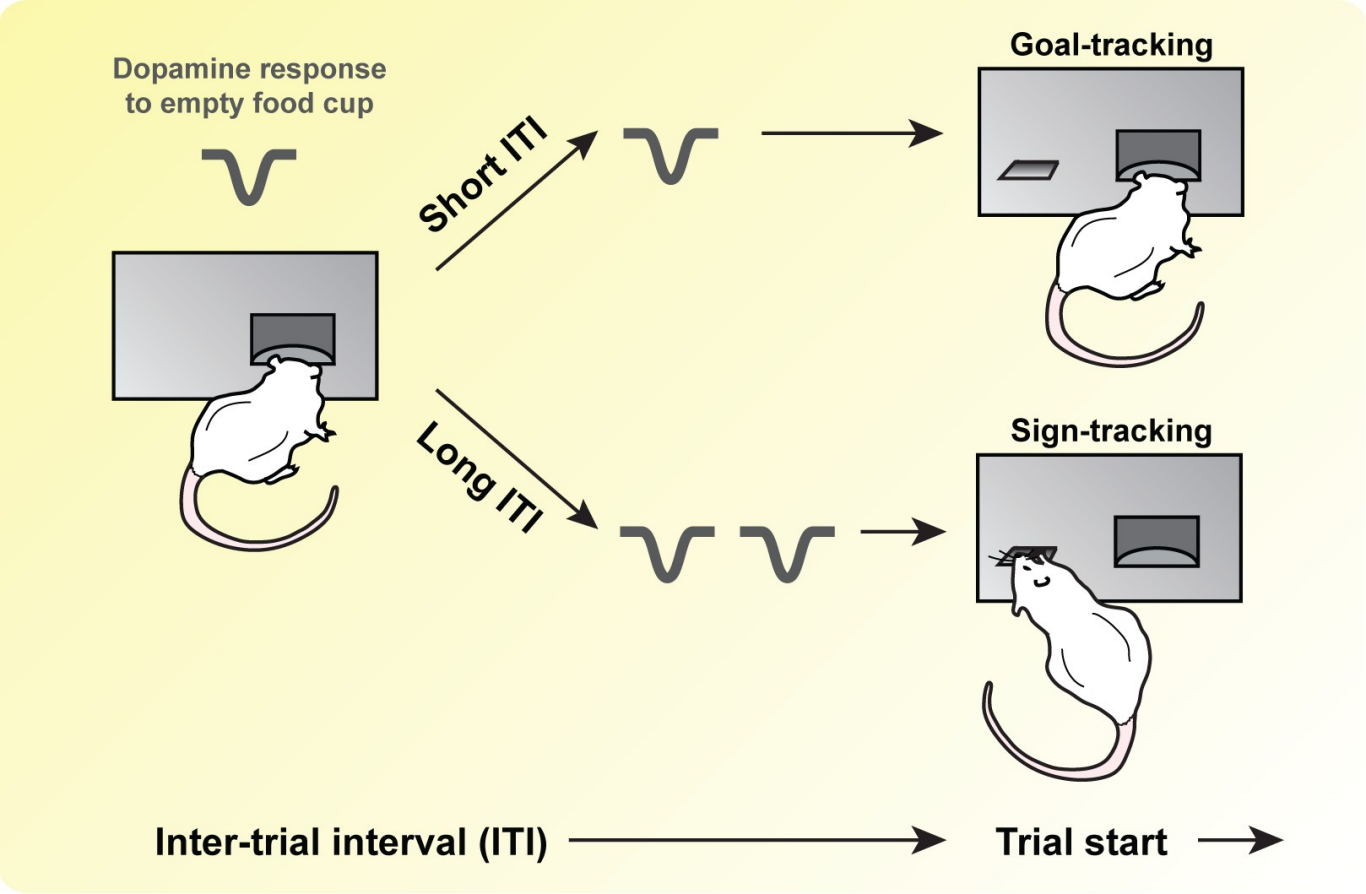
Experimental predictions. In this issue of *PLOS Biology*, Lee and colleagues randomized rats to short or long ITIs and measured behavior and dopamine release. They predicted that with a long ITI, rats would have more opportunities to engage with an empty food cup, causing repeated negative dopamine responses and biasing the animals toward a model-free, sign-tracking approach. In contrast, short ITIs would bias the animals toward a model-based, goal-tracking approach. ITI, intertrial interval.

The experiment was simple: on each trial, a lever was presented for 8 s, followed by pellet delivery into the food cup. Some animals then waited 60 s for the next trial to begin, while others had to wait 120 s. As expected, Lee and colleagues found that rats randomized to the 120-s ITI were more likely to sign-track (i.e., approach the lever), while rats randomized to the 60-s ITI were more likely to goal-track (i.e., approach the food cup). This was particularly apparent at the beginning of each trial; after 4 s, the two groups behaved similarly. While the rats were performing the task, the authors used fast-scan cyclic voltammetry to measure a proxy of DA release in the nucleus accumbens and found that the long-ITI group showed significantly higher DA release to both the lever and the food cup compared to the short-ITI group. In fact, the short-ITI group did not show any significant DA response to the reward after learning. These results correlated with the amount of time the rats interacted with the food cup during the ITI: the more time rats spent with the food cup during the ITI, the higher the DA release to both the lever and the food cup. These changes in behavior and DA release developed over a similar time course, implying that they either drive each other or that they are both driven by a common factor.

Together, Lee and colleagues show that a simple manipulation—changing the amount of time between trials—can modulate behavior and DA release in the nucleus accumbens. Based on the STGT model, the authors interpret their results as reflecting interactions with an empty food cup between trials, triggering a lower estimate of the food cup’s value, and biasing the rats toward a model-free, sign-tracking approach. Curiously, when the rats were nudged toward sign-tracking behavior, their DA responses did not recapitulate the sign-tracking pattern observed by Flagel and colleagues [14], with reward responses decreasing over time. Similarly, when the rats were nudged toward goal-tracking behavior, the DA responses did not show the sustained reward response one might have expected from pure goal-trackers [14]. In both cases, DA release was a hybrid of the two patterns. This demonstrates that sign tracking and goal tracking are not hardwired and unchanging; rather, these tendencies arise from specific interactions the animals have with stimuli in a task. By the same logic, it is unlikely that the DA-dependent model-free system is ever completely offline; instead, animals give this system more or less weight depending on their experience and needs. The simple one-to-one relationship between DA release and behavioral strategy turns out to be much more complex.

These results suggest a number of important future directions. For example, to shore up the correlations between food cup interactions and DA responses (Fig 2), investigators might consider removing the food cup entirely during the ITI [21]. If the STGT interpretation is correct, this should abolish any effect of ITI on the rats’ behavior. If behavioral differences remain, other interpretations must be sought—for example, long waiting times might simply increase the salience of all stimuli, regardless of interactions with those stimuli in the meantime. Another fruitful approach might be a cross-over design, in which rats experience both ITI lengths at different points in the experiment. Is the effect strong enough to shift an individual rat back and forth between sign tracking and goal tracking? Higher resolution, trial-by-trial measurements of DA (e.g., through extracellular recording) might also be crucial to explore the complicated temporal dynamics of behavior on this task. Finally, causal manipulations of the DA circuit, especially through temporally specific means such as optogenetics, would go a long way toward demonstrating that patterns of DA activity actually control behavior in this task, rather than simply reflect it.

For the field more generally, numerous fascinating questions remain open. Where in the brain do model-based and model-free learning occur, and what determines the relative weight between them [19]? How are RPEs calculated in the first place [22]? Is DA the only relevant circuit for RPE-driven model-free learning, or are there redundant circuits, for example, in the cerebellum [23]? What are the genetic or environmental factors that bias some animals to sign-track and others to goal-track under equivalent testing conditions? What relationship does this phenomenon have to disorders of learning or habit, such as OCD or addiction? Finally, emerging evidence from multiple species indicates that a subset of DA neurons is activated by unpleasant or painful stimuli [24–26]. Is there undiscovered individual variability in behavior or DA activity within the aversive domain? Should aversive DA signals be integrated into computational frameworks for reward learning, or do these represent entirely separate computations? These questions will take concerted effort across many labs and multiple years, but they may hold the key to some of the most fundamental questions in neuroscience: how we learn about rewards and punishments and how this process breaks down in neuropsychiatric disease.

Zooming out even further, it is important to note Lee and colleagues’ general approach. They studied a computational model of an interesting physiological phenomenon, developed a specific hypothesis from that model, and then collected new data to test this hypothesis directly. What they found partially validated the model but also brought up new and unanticipated questions. Regardless of the specific results, this is the scientific method at its purest, and it should be celebrated.

## Abbreviations

DA: dopamine
ITI: intertrial interval
STGT: Sign Tracking and Goal Tracking
RPE: reward prediction error
